# Recognizing emotions in music through a computerized method: a novel way of evaluating social maturity

**DOI:** 10.3389/fpsyt.2025.1674615

**Published:** 2025-10-17

**Authors:** Hyunchan Hwang, Jin Hyung Lee, Juri Yun, Seo-Koo Yoo, Doug Hyun Han

**Affiliations:** ^1^ Department of Psychiatry, College of Medicine, Chung-Ang University, Seoul, Republic of Korea; ^2^ Master of Arts in Music Therapy, Faculty of Fine and Applied Arts, Chulalongkon University, Bangkok, Thailand; ^3^ Center of Excellence: FAA-EMILI Sagol Creative Arts Research and Innovation for Well-being Center, Chulalongkorn University, Bangkok, Thailand; ^4^ Department of Music Therapy, Ewha Womans University, Seoul, Republic of Korea; ^5^ School of Social Welfare, Soongsil University, Seoul, Republic of Korea

**Keywords:** emotion perception, social maturity, music emotion, facial emotion recognition, autism spectrum disorder

## Abstract

**Introduction:**

This study used music emotion recognition to evaluate emotion perception and aimed to explore the relationship between social maturity and musical emotion recognition in both autism spectrum disorder (ASD) and controls groups.

**Methods:**

84 people with ASD and 50 controls were included in the study. Participants were evaluated using the Wechsler Intelligence Scale of corresponding age (IQ), social maturity scale (SQ), childhood autism rating scale 2 (CARS). Emotion perception was evaluated using two tools. The emotion perception test (EPT) shows multiple facial expressions and asks to find the face with a different emotion than the rest. The music emotion recognition test (MEPT) asks the user to choose the emotion they feel when they hear the given music.

**Results:**

The control group had significantly higher IQ, SQ, EPT scores, MEPT scores, and significantly lower CARS scores. Since IQ differences were substantial between the two groups, individuals with IQ lower than 70 were removed from the main analysis, and are not interpreted causally, but only presented as associations. SQ was positively correlated with EPT scores in all participants and ASD group, but not in the control group. Correlation between MEPT scores and SQ were also similar to that of EPT scores and SQ.

**Discussion:**

The MEPT shows that social intelligence as music emotion recognition are correlated. Further research may prove this tool to be useful for people with ASD.

## 1 Introduction

Emotion—how it is defined, evoked, perceived, or understood—has been a topic of scientific interest since the age of Aristotle (1). Giants in the field such as Charles Darwin (2) and William James (3) have left their mark (4), but much more research is needed to fully understand emotions. One consensus, however, is that the ability to recognize emotions is one of the key bases of social cognition (5), which is how we perceive, process, interpret, and respond to social stimuli (6).

Many factors are involved in emotional perception and social cognition, including age (7), gender (8), and intelligence (9). Additionally, various psychiatric illnesses have been linked to deficits in social cognition. Schizophrenia patients have shown poorer facial recognition abilities compared to controls (10), and the decline of social functions in the chronic phase of the disease has been well documented (11). Personality disorders such as borderline personality disorder and antisocial personality disorder are also associated with deficits in perceiving social cues (12) and recognizing emotions in faces (13). However, it can be argued that autism spectrum disorder (ASD) is unique in the social cognition difficulties that it poses (14).

Although there are many factors associated with emotion recognition in ASD, such as sex (15, 16), intelligence (9, 16), and age (9, 16), these results might not be consistent, as prior studies often have had conflicting data (17). However, there is a considerably stronger consensus about social intelligence’s relationship with emotion recognition (18, 19), and many researchers have studied emotion perception in ASD. Weight et al. (20) has noted that people with ASD have difficulty in face memory and face perception tasks. One recent meta-analysis by Yeung has highlighted impaired facial emotion recognition in ASD in various settings (21). Of the few studies that reported differences in reaction time in facial emotion recognition tests, Homer et al. (22) showed that people with ASD exhibited longer response times compared to controls, and Wagener et al. (23) found that more severe autism was correlated with longer reaction time. However, there are also reports of reaction times having no difference between ASD and control groups, showing the need for further research (24, 25). There is also some evidence that in ASD there are deficits associated with the functioning of the fusiform gyrus, which is crucial for identifying facial features (26). Dziobek et al. (27) also found through a study comparing age-, sex-, and IQ-matched participants with ASD and neurotypical controls that the former performed worse at recognizing facial emotions and that this was negatively correlated with fusiform gyrus thickness.

These data all suggest some kind of deficit with facial recognition in ASD individuals, placing them at a disadvantage in evaluating emotional perception through facial stimulation. Also, some researchers have shown that there are differences in amygdala function between people with ASD and controls (28), indicating that emotion perception difficulties may appear in domains other than facial expressions. Due to these limitations of facial emotion recognition, many researchers have searched for alternative methods to evaluate emotion recognition (29, 30). One of these methods is emotion recognition through music.

How this music-driven emotion recognition would manifest in ASD individuals has also been a topic of interest. Some researchers have speculated that emotion perception through music is intact in ASD (31). Indeed, there have been reports of people with ASD having preference for musical stimuli over verbal ones and cases of ASD individuals showing better music processing abilities than typically developing peers (32). Researchers advocating for this usually reference the shared affective motion experience model, which posits that that music is perceived not only by auditory signals but also by coordinated sequences of motor activities (33). Also, using music for psychological evaluation may increase engagement, which could result in more accurate test scores. Low engagement in tests can make it difficult to truly evaluate the individual’s ability (34). Using music to increase engagement has already been used in teaching settings (35), and incorporating this into psychological tests could be useful. Evaluating ASD people using music may also have downsides, as 40~90% of ASD people have shown hypersensitivity to various stimuli, including auditory ones (36, 37). However, Bhatara et al. (38) reported that although ASD children experience more frequent hypersensitivity when compared to typically developing (TD) peers, this does not affect their enjoyment of music. Also one review on the literature found that most causes of hypersensitivity in ASD were sounds like vacuum cleaner, sirens, machines, etc and no references to music related discomfort was found (39).

Nevertheless, few studies attempt to assess the emotion recognition abilities of ASD people through music, with many of them lacking in sample size or study design (31, 40). The few studies with sufficient sample size and sound design have yielded mixed results. For instance, Bhatara et al. (41) found impairment in judging the expressiveness of emotion in music among people with ASD compared to controls matched on performance IQ and auditory working memory. In contrast, Quintin et al. (42) found no differences in music emotion recognition between high-functioning ASD individuals and controls when controlling for verbal IQ.

Therefore, we developed a tool, named Music Emotion Perception Tool (MEPT) to evaluate emotion perception through recognition of emotional expression in music and compared it with standardized facial emotion perception tools such as the Emotion Perception Test (EPT) in both the ASD group and healthy controls. We hypothesized that the MEPT would demonstrate acceptable internal consistency, construct validity and concurrent validity. Also, we hypothesized that the MEPT scores would be significantly different lower in the ASD group when compared to the TD group. Finally, we hypothesized that MEPT scores would be significantly correlated with social intelligence performance.

## 2 Materials and methods

### 2.1 Participants and procedure

Two groups of participants (people with ASD and TD controls) were recruited between October 2020 and September 2022. The first group consisted of adolescents and adults diagnosed with ASD. Those included in the ASD group (1) were between 13 and 35 years old, (2) had been diagnosed with autism spectrum disorder based on the Diagnostic and Statistical Manual of Mental Disorders-5 (43) criteria, and (3) agreed to participate in the study of their own accord. The control group consisted of TD adolescents and adults who (1) were between 13 and 35 years old, (2) had not been diagnosed with autism spectrum disorder, intellectual developmental disorder, or any other developmental disorder, and (3) agreed to participate in the study of their own accord. The exclusion criteria for the two groups were (1) inability to understand or follow the research protocol, (2) inability to understand the Korean language, (3) diagnosis with another major psychiatric or neurological disorder, and (4) hearing difficulties.

In total, there were 84 participants in the ASD group and 50 participants in the TD (control) group. Level 3 ASD participants were excluded due to their inability to follow the research protocol without very substantial support. Most participants in the ASD group had level 1 ASD. The participants were assessed at Chung-Ang University Hospital. They visited the hospital twice. During the first visit, their eligibility for the study was assessed, and baseline demographic information, IQ, and Childhood Autism Rating Scale 2 scores were collected. If deemed eligible for the study, participants returned to the hospital to complete the other remaining tests.

### 2.2 Ethics approval and consent to participate

All participants involved in the research provided written informed consent approved by the Institutional Review Board of Chung-Ang University (1041078-202008-HRBM-235-01, 1041078-202108-HRBM-266-01). For minors or participants diagnosed with intellectual developmental disorder, the legal guardian was also informed of the study procedures and provided written informed consent along with the participant. All actions have been performed in accordance with the declaration of Helsinki.

### 2.3 Assessments

#### 2.3.1 Demographics

Participants reported their age, gender, years of education, and economic status. Years of education referred to the number of years that participants had been attending school, beginning with the first year of elementary school. For economic status, participants were asked to write their approximate monthly income of the whole immediate family.

#### 2.3.2 Intelligence assessment

Intelligence was evaluated using the Korean Wechsler Intelligence Scale for Children IV (K-WISC-IV) (44) or the Korean Wechsler Adult Intelligence Scale IV (K-WAIS-IV) (45) for the relevant age groups. These are standardized tests measuring individuals’ cognitive ability, with a population mean score of 100. The full set Intelligence Quotient (IQ), along with the 4 subscales (Verbal comprehension, Perceptual organization, Working memory, Processing speed) were analyzed in our study. Because the age group eligibility for the two tests overlapped at age 16, participants of that age completed the K-WAIS-IV.

#### 2.3.3 Korean childhood autism rating scale 2

The Korean Childhood Autism Rating Scale 2 (K-CARS2) (46) is a standardized assessment tool to identify children with ASD aged 2 years and older and determine autism severity. Although the test can be conducted through observation of the participant alone, the clinical psychologist in this study also interviewed the parent in person or over the phone, as most participants could not remember how they behaved when they were very young.

#### 2.3.4 Social maturity scale

The Social Maturity Scale (SMS) (47) was used to evaluate participants’ social maturity. This tool has been adapted and standardized from the Vineland Social Maturity Scale (48) for the Korean population (47). Using this tool, clinical psychologists can assess individuals’ social competence by examining variables such as self-direction, locomotion, occupation, communication, self-help, and socialization. After evaluation, the social quotient (SQ) was calculated by dividing the social age by the current age and multiplying it by 100, according to the manual provided by the scale developers (47).

#### 2.3.5 Korean version of center for epidemiologic studies depression scale-revised

The Korean version of Center for Epidemiologic Studies Depression Scale-Revised (CES-D) (49) is a self-report scale developed to evaluate depressive symptoms. The scale consists of 20 questions that asks the user on various depression related symptoms on a 5-point Likert scale. The scale has been revised according to the Diagnostic and Statistical Manual of Mental Disorders-IV (50), and is widely used to assess depressive symptoms in various settings.

#### 2.3.6 Korean beck anxiety inventory

The Korean Beck Anxiety Inventory (BAI) (51) was selected for evaluation of anxiety symptoms in our study. The scale is a self-report scale consisting of 21 items on anxiety, and each item is measured on a four-point Likert scale. The BAI was designed to rate anxiety separate from confounding depressive symptom, and has shown to be a valid way to measure anxiety (52).

#### 2.3.7 Emotion perception test

The EPT was designed by Bae et al. (53) to evaluate individuals’ mood status. Participants encountered 2–8 standardized faces per question, and they were instructed to choose whether the faces all showed the same emotion, or they showed different emotions. Correction rate and response time for all 108 questions were calculated at the end of the test. The emotions conveyed by the faces were normed by 208 individuals living in the Republic of Korea, the location of the current study (53). The EPT has been also used to test facial emotion recognition in schizophrenic and bipolar patients (10).

#### 2.3.8 Music emotion perception test

The Music Emotion Perception Test (MEPT) was developed by the current research team as an alternative tool to assess individuals’ abilities to perceive and differentiate emotions expressed in music. Unlike traditional measures that rely heavily on facial or visual cues, the MEPT emphasizes auditory stimuli, specifically musical excerpts. This is especially relevant for individuals with autism spectrum disorder (ASD), who often demonstrate reduced attention to faces and may process emotional information more effectively through auditory channels than through visual ones. The initial development and validation of this measure were published previously (54). For the sake of transparency, clarity, and replicability, we summarize the methodological details in full here.

##### 2.3.8.1 Stimulus development

Stimulus creation was undertaken in a carefully staged process to ensure both ecological validity and psychometric rigor. The research team initially identified 120 instrumental musical excerpts, evenly divided into three target emotional categories: happiness, sadness, and anger (40 per category). Three researchers trained in music therapy carried out this initial classification by drawing on both the existing literature on music and emotion perception and their own clinical and musical expertise.

Several strict selection criteria were applied. First, to minimize linguistic and cultural bias, all excerpts were purely instrumental and contained no lyrics or vocalizations. Second, passages were limited to a maximum of two instruments to control for excessive textural complexity. This ensured that emotional perception was not confounded by the number of simultaneous musical lines. Common instrumentations included piano solos, guitar solos, and string–piano combinations. Percussion instruments were excluded, since their strong rhythmic drive can dominate listeners’ attention and complicate emotion appraisal.

Excerpts were standardized to 15 seconds in duration, following evidence that this length is sufficient to convey emotional qualities while avoiding problems associated with emotional shifts across longer excerpts. For five particular pieces where the intended emotional character did not emerge until later in the passage or where there was a sudden shift in affective content, the starting point was adjusted so that the 15-second excerpt represented a stable and unambiguous emotional state. Genres represented in the final pool included Western classical, new age, and jazz, reflecting both the traditions most frequently used in prior music emotion recognition (MER) research and the availability of emotionally distinct passages.

##### 2.3.8.2 Expert ratings

In the first validation phase, a panel of 20 certified music therapists evaluated the 100 shortlisted passages. Demographic data indicated an average age of 32.6 years, with 95% of the sample female, and a mean of approximately 11 years of formal musical training. Each therapist rated the appropriateness of the researcher-assigned emotion label (happy, sad, or angry) as well as the perceived expressive intensity of the excerpt on a 7-point Likert scale. This dual rating procedure allowed the team to capture not only categorical agreement but also gradations of expressivity.

From these expert ratings, the 20 excerpts with the highest intensity and lowest disagreement within each emotional category were retained. The resulting set of 60 passages (20 happy, 20 sad, 20 angry) became the foundation of the MEPT. This process provided strong content validity, as the final stimuli reflected both theoretical criteria and consensus judgments from clinical professionals.

##### 2.3.8.3 Validation sample and procedure

The second phase tested the MEPT on a large validation sample of 300 neurotypical adults (mean age 29.2 years; 41% male). Recruitment occurred via online postings on university boards, social media groups, and community forums. Inclusion criteria required participants to be between 18 and 40 years of age, while exclusion criteria ruled out individuals with professional musical training, hearing impairments, developmental disabilities, or psychiatric history.

Participants completed the task through the SurveyMonkey platform, where the 60 musical excerpts were embedded via SoundCloud streaming links. The presentation order was randomized for each participant to minimize sequence effects. For each excerpt, participants were asked to complete two judgments: 1) Categorical classification — selecting whether the excerpt expressed happiness, sadness, or anger; 2) Intensity rating — rating the strength of the expressed emotion on a 7-point Likert scale, ranging from “extremely weak” to “extremely strong”.

Responses were then coded against the expert-determined labels. A correct classification was scored as “1,” and an incorrect classification as “0,” producing a total possible score of 0–60. This binary scoring system provided an objective performance measure while also allowing separate analysis of intensity ratings.

##### 2.3.8.4 Psychometric properties

Statistical analyses indicated that the MEPT possesses excellent reliability and validity. Internal consistency was extremely high, with Cronbach’s α = 0.978 across the 60 items. Exploratory factor analysis confirmed the hypothesized three-factor structure corresponding to happiness, sadness, and anger (Kaiser-Meyer-Olkin = 0.969; Bartlett’s χ² = 12,996.76, p < 0.001).

Cluster analysis further revealed a robust division between participants with higher and lower emotion perception ability, yielding a cut-off score of ≤41 to identify individuals with low performance (F = 1,120.63, p < 0.001). Hierarchical logistic regression analyses showed that only MEPT performance scores, not demographic or psychological factors such as age, gender, mood, or anxiety levels, predicted membership in the low-perception group (Nagelkerke’s R² = 0.735). This suggests that the MEPT captures a domain-specific skill in emotion perception that is not confounded by general psychological or demographic variables.

##### 2.3.8.5 Availability and replicability

All 60 validated excerpts are documented in detail in the Supplementary Tables of Lee et al. (2023). These include information on title, composer, instrumentation, and excerpt start times, allowing independent researchers to recreate the exact set of stimuli. Audio files are accessible through commercially available recordings or by request from the authors. The combination of transparent stimulus documentation, clear scoring rules, and reported validation parameters ensures that the MEPT can be independently replicated without reliance on unpublished or proprietary materials.

### 2.4 Statistical analysis

Independent t-tests and chi-square tests were done to compare baseline demographic, psychological, and other variables between the ASD group and neurotypical controls. To calculate the internal consistency of the MEPT, tetrachoric correlation was chosen to calculate the ordinal alpha, as it was deemed to more accurately estimate the relationship between the variables (55). Exploratory factor analysis (EFA) was performed for the evaluation of construct validity of the MEPT. In addition, the EFA was conducted three times to assess the construct validity of the three substructures of MEPT. The eigenvalues were calculated for each item, and those with factor loadings lower than 0.4 were considered unstable and therefore statistically insignificant (56) (**Supplementary 1**). For evaluation of the concurrent validity of MEPT, pearson correlation coefficient was calculated between MEPT and EPT.

Shapiro-Wilk tests were done to check for normality. Independent t test was done for all continuous variables and chi square was done for ordinal and categorical variables such as SES and gender. In the case of non-normality, Mann-Whitney U test was performed. To account for the effect of IQ on SQ and EPT, MEPT, univariate ANCOVA with IQ as the covariate was also performed. We evaluated homogeneity of regression slopes by testing the Group × IQ interaction, and in the case the group x IQ interaction was significant, we probed conditional group differences with linear regression models including Group, IQ, and Group × IQ, re-centering IQ at prespecified values within the observed overlap of the two groups’ IQ distributions. Analysis based on age group (Adolescent=13~18, Adult=19~33) was also tested for interaction effects. Partial pearson coefficient was also calculated to evaluate the correlation between SQ and MEPT, EPT with IQ as a covariate.

Even with these statistical adjustments, it was deemed that the IQ difference between the two groups were too high. Therefore, IQ under 70 was removed for the main analysis, and 43 out of the 84 ASD group participant were analyzed along with the 50 participants in the control. Both analysis of 84 ASD participants, and 43 ASD participants without intellectual disabilities are provided separately. To account for multiple comparison, Bonferroni correction was performed and to account for the 7 univariate ANCOVA and 9 partial correlation performed, p=0.05/16 = 0.003 was seen as significant for the univariate ANCOVA and partial correlation. Except for tetrachoric correlation, which was conducted using R statistics 4.1.1, all other analyses were conducted using SPSS 24.

## 3 Results

### 3.1 Demographics

There were no significant differences in age, gender, or economic status between participants with ASD and neurotypical controls. However, ASD participants had fewer years of education, lower SQ and IQ, and higher scores on CARS, BAI, and CES-D compared to controls (**Table 1**).

**Table 1:** Demographic and clinical characteristics.

Variables	ASD (n=84)	Control (n=50)	Statistical value	95% Confidence interval
Age, years (Mean ± SD)	21.07 ± 3.84	22.04 ± 3.75	t=1.43 p=0.16	[-0.38, 2.31]
Gender, number of male/female^†^	58/26	32/18	χ^2^ = 0.36 p=0.57	
Years of education, years (Mean ± SD)^*^	11.51 ± 2.47	13.84 ± 2.63	t=5.15 p<0.001	[1.43, 3.22]
Economic status (family income per month) ^†^				
Under 2 million Korean Won	18 (21.4%)	13 (26.0%)		
2 ~ 4 million Korean Won	56 (66.7%)	28 (56.0%)	Linear χ^2^ = 0.20 p=0.89	
Over 4 million Korean Won	10 (11.9%)	9 (18.0%)		
IQ Total, FSIQ (Mean ± SD) ^*^	71.34 ± 19.69	107.37 ± 13.88	t=12.38 p<0.001	[30.28, 41.79]
Verbal comprehension, index score (Mean ± SD) ^*^	80.36 ± 17.14	105.21 ± 12.13	t=9.79 p<0.001	[19.83, 29.88]
Perceptual organization, index score (Mean ± SD) ^*^	79.29 ± 19.97	107.69 ± 15.21	t=9.27 p<0.001	[22.33, 34.45]
Working memory, index score (Mean ± SD) ^*^	75.31 ± 20.15	108.40 ± 15.52	t=10.65 p<0.001	[26.94, 39.24]
Processing speed, index score (Mean ± SD) ^*^	69.67 ± 15.87	103.14 ± 15.66	t=11.87 p<0.001	[27.89, 39.05]
Childhood Autism Rating Scale-2, t score (Mean ± SD) ^*,††^	38.30 ± 12.73	20.02 ± 0.14	t=-13.01 p<0.001	[-21.08, -15.49]
Beck Anxiety Inventory (Mean ± SD) ^*^	14.25 ± 12.44	4.18 ± 3.67	t=-6.93 p<0.001	[-12.95, -7.19]
Center for Epidemiologic Studies Depression Scale-Revised (Mean ± SD) ^*^	22.95 ± 10.18	19.74 ± 4.90	t=-2.09 p=0.04	[-6.25, -0.17]

ASD, Autism spectrum disorder; SD, Standard deviation; IQ, Intelligence quotient; FSIQ, Full scale intelligence quotient; *p=0.05 was considered significant; †Chi-square was used for analysis. Independent t-test was used for all other analyses; ††2 values in the ASD group were missing.

### 3.2 Power analyses

In general, the recommended sample size for EFA is at least 5 to 10 respondents per item (57). In this study, the number of items in the MEPT substructures ranged from 7 to 17, meaning that the required number of participants should be between 85 and 170. Therefore, with 134 participants, we considered the sample size to be appropriate for EFA.

In correlation analysis, the sample size was calculated with 128 as the number of subjects (power = 0.97, effect size=0.3, α =0.05). In t-test analysis, the sample size was calculated with 126 as the number of subjects (power = 0.87, effect size=0.5, α =0.05).

### 3.3 Internal consistency

The full MEPT, consisting of 39 total items (MEPT-1: 21 items, MEPT-2: 9 items, MEPT-3: 9 items), demonstrated evidence of internal consistency, with a tetrachoric ordinal of 0.951. This was also the case for the MEPT-1, MEPT-2, and MEPT-3 with tetrachoric ordinal alphas of 0.873, 0.850, and 0.960, respectively.

### 3.4 Construct validity

In the EFA, the 17 items with a factor loading of 0.4 or higher on the MEPT-1 were selected. For the MEPT-1, factor loading of items 8, 12, 15, and 21 were under 0.4. The factor loading of 7 items with a factor loading of 0.4 or higher on the MEPT-2 were selected. For the MEPT-2, the factor loading of items 6 and 9 were under 0.4. The factor loading of all items on the MEPT-3 were 0.4 or higher. In the EFA conducted with 33 items (39 minus 6 items with low factor loading), the factor loading of all items were 0.4 or higher.

### 3.5 Concurrent validity

For all participants, the EPT correction rate was positively correlated with MEPT-1 (r = 0.790, p < 0.001) and MEPT-2 (r = 0.585, p < 0.001) correction rates. Although MEPT-3 (r = 0.643, p < 0.001) also showed significant correction rates, due to the high ceiling effect, the construct and concurrent validity of MEPT-3 was deemed unacceptable. Analysis of MEPT-3 was therefore retained as exploratory and was removed from the main analysis.

### 3.6 Comparison of SQ, EPT and MEPT scores between ASD and control groups

The ASD group showed decreased scores for the SQ scores and EPT correction rate compared to the control group. However, there was no significant difference in reaction time between the two groups (**Table 2**).

**Table 2:** Differences between social maturity, EPT and MEPT.

Variables	ASD (n=43)	Control (n=50)	Statistical value	95% Confidence interval
Intelligence quotient, raw score (Mean ± SD) ^*^	87.87 ± 11.25	107.37 ± 13.88	t=7.48 p<0.001 d=1.54	[14.32, 24.68]
Social quotient, raw score (Mean ± SD) ^*^	67.10 ± 17.47	105.77 ± 10.30	t=12.74 p<0.001 d=2.70	[32.61, 44.74]
Emotion Perception Test				
Correction rate, score/total question (Mean ± SD) ^*^	0.66 ± 0.16	0.84 ± 0.06	t=7.06 p<0.001 d=1.49	[0.13, 0.23]
Reaction time, millisecond (Mean ± SD)	4,009 ± 1,297	4,108 ± 1,024	t=0.41 p=0.68 d=0.08	[-379, 578]
Music Emotion Perception Test				
MEPT-1 correction rate, raw score (Mean ± SD) ^*^	14.47 ± 3.67	18.30 ± 1.57	t=6.39 p<0.001 d=1.36	[2.63, 5.04]
MEPT-1 reaction time, millisecond (Mean ± SD)	2,427 ± 1,297	2,425 ± 1,609	t=-0.07 p=0.995 d<0.01	[-602, 598]
MEPT-2 correction rate, raw score (Mean ± SD) ^*^	5.74 ± 1.95	7.48 ± 1.05	t=5.21 p<0.001 d=1.11	[1.07, 2.40]
MEPT-2 reaction time, millisecond (Mean ± SD)	3,689 ± 1,966	3,343 ± 1,646	t=-0.93 p=0.36 d=0.19	[-1,090, 398]

ASD, Autism spectrum disorder; SD, Standard deviation; EPT, Emotion perception test; MEPT, Music emotion perception test; d, cohen’s d; *p=0.003 was considered significant; Independent t-test was done.

The ASD group showed decreased scores on all MEPT sub-tests compared to the control group (**Table 2**). There were no significant differences in reaction times on any sub-tests between the two groups. However, Shapiro–Wilk test showed that p<0.05, therefore non-normality of the data was assumed. Since there were no outliers visible, Mann-Whitney U test was also done on the reaction times of all MEPT. Results showed that MEPT-1 (U = 993.00, Z=-0.63, p=0.53), MEPT-2 (U = 967.00, Z=-0.83, p=0.41) were also non-significant.

When adjusted for IQ, preliminary checks for homogeneity of regression slopes were met for SQ (F = 1.79, p=0.19, partial η²=0.02), EPT scores (F = 0.11, p=0.74, partial η²=0.001), EPT reaction time (F = 1.91, p=0.17, partial η²=0.02), MEPT-1 scores (F = 0.17, p=0.68, partial η²=0.002), MEPT-1 reaction time (F = 0.04, p=0.84, partial η²<0.001), MEPT-2 scores (F = 0.80, p=0.37, partial η² =0.009), MEPT-2 reaction time (F = 0.08, p=0.78, partial η²=0.001), indicating interaction between group and IQ was not significant. Since Levene’s test was significant for SQ, EPT scores, MEPT-1 scores and MEPT-2 scores, bootstrapping with 1,000 samples was used for those variables (**Table 3**).

**Table 3:** Differences between social maturity, EPT and MEPT with IQ as covariate.

Variables	Adjusted ASD (n=43)	Adjusted Control (n=50)	Statistical value	95% Confidence interval
Social quotient, raw score (Mean ± Standard error) ^*^	62.81 ± 15.52	105.77 ± 10.30	F=83.34 p<0.001 η²=0.48	[25.44, 39.60]
Emotion Perception Test				
Correction rate, score/total question (Mean ± Standard error) ^*^	0.67 ± 0.02	0.83 ± 0.02	F=26.41 p<0.001 η²=0.23	[0.09, 0.22]
Reaction time, millisecond (Mean ± Standard error)	3,915 ± 203	4,189 ± 185	F=0.81 p=0.37 η²=0.009	[-331, 879]
Music Emotion Perception Test				
MEPT-1 correction rate, raw score (Mean ± Standard error) ^*^	14.89 ± 0.47	17.94 ± 0.43	F=18.38 p<0.001 η²=0.17	[1.64, 4.46]
MEPT-1 reaction time, millisecond (Mean ± Standard error)	2,322 ± 255	2,516 ± 232	F=0.26 p=0.61 η²=0.003	[-565, 954]
MEPT-2 correction rate, raw score (Mean ± Standard error) ^*^	6.02 ± 0.26	7.25 ± 0.24	F=9.66 p=0.003 η²=0.10	[0.44, 2.02]
MEPT-2 reaction time, millisecond (Mean ± Standard error)	3,835 ± 316	3,218 ± 288	F=1.70 p=0.20 η²=0.02	[-1,425, 283]

ASD, Autism spectrum disorder; SD, Standard deviation; EPT, Emotion perception test; MEPT, Music emotion perception test; d, cohen’s d; *p=0.003 was considered significant; Univariate ANCOVA with IQ as covariate; bootstrap (1,000) used when Levene’s test significant.

**Figure 1:**
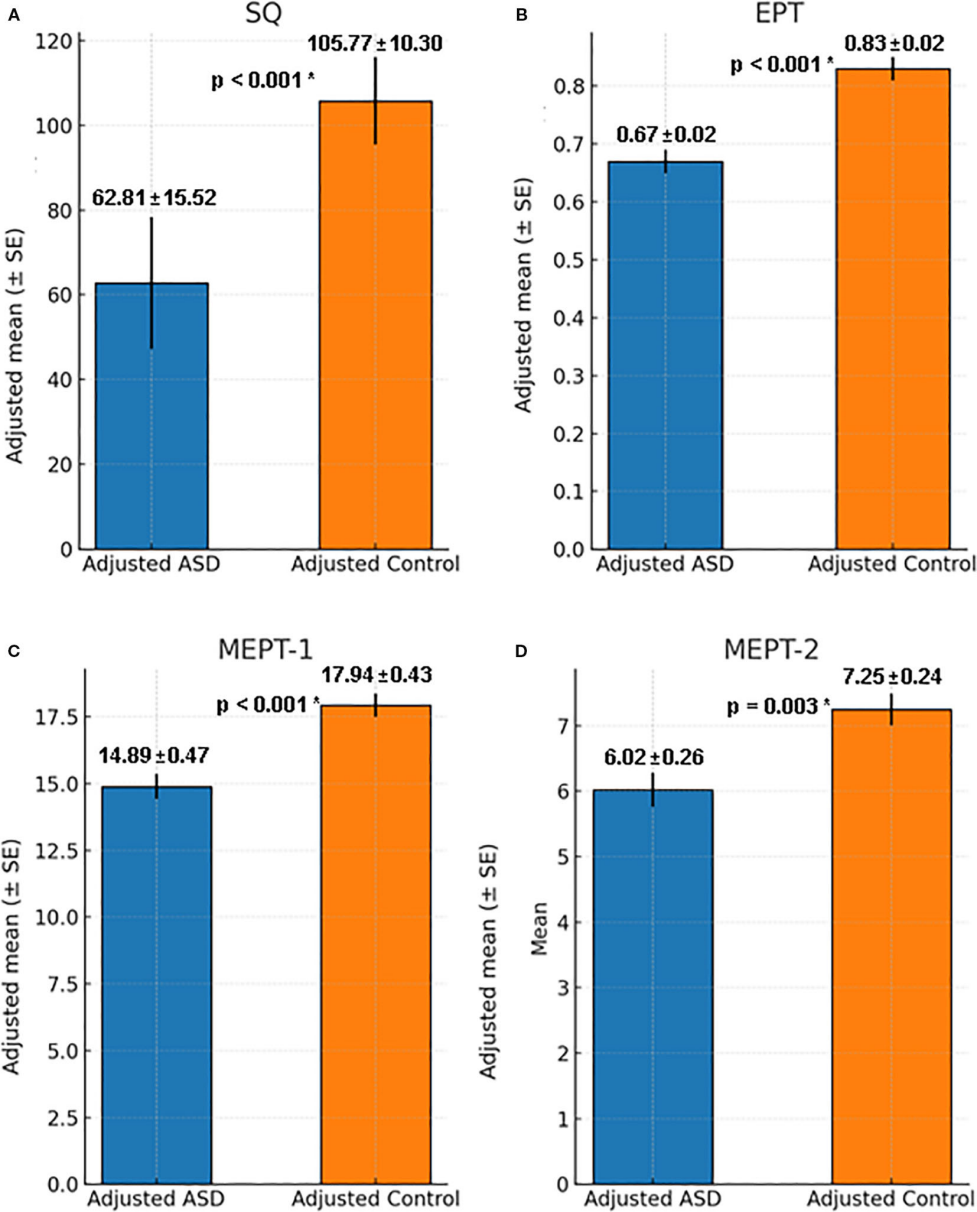
Comparison of Adjusted SQ, EPT and MEPT between ASD group and control group with IQ as covariate. **(A)** Social quotient for ASD and control group when adjusted for IQ, F = 83.34, p < 0.001, η²=0.48, 95% Confidence Interval [25.44, 39.60]. **(B)** EPT score for ASD and control group when adjusted for IQ, F = 26.41, p < 0.001, η²=0.23, 95% Confidence Interval [0.09, 0.22]. **(C)** MEPT-1 score for ASD and control group when adjusted for IQ, F = 18.38, p < 0.001, η²=0.17, 95% Confidence Interval [1.64, 4.46]. **(D)** MEPT-2 score for ASD and control group when adjusted for IQ, F = 9.66, p = 0.003, η²=0.10, 95% Confidence Interval [0.44, 2.02]. ASD, Autism spectrum disorder; SQ, Social quotient; EPT, Emotion perception test; MEPT, Music emotion perception test; *p=0.003 was considered significant.

IQ adjusted ASD and controls had significant differences in social quotient (F = 83.34, p<0.001, partial η²=0.48), EPT scores (F = 26.41, p<0.001, partial η²=0.23), MEPT-1 scores (F = 18.38, p<0.001, partial η²=0.17) and MEPT-2 scores (F = 9.66, p=0.003, partial η²=0.10). However, there were no significant differences in EPT reaction time (F = 0.81, p=0.37, partial η²=0.009), MEPT-1 reaction time (F = 0.26, p=0.61, partial η²=0.003) and MEPT-2 reaction time (F = 1.70, p=0.20, partial η²=0.02) (**Figure 1**).

Analysis on the whole test group also showed similar results with and without adjusting for IQ. The results of the analysis can be seen in the supplements (**Supplementary 2**).

Interaction effects between age group (adolescent vs adult) showed non-significance for SQ, EPT and MEPT scores. Detailed results on the effects of age group can also be seen in the supplements (**Supplementary 3**).

### 3.7 Correlations between SQ scores and EPT scores

For all participants, SQ scores were positively correlated with the EPT correction rate after adjusting for IQ (partial r=0.64, p<0.001, CI [0.46, 0.77]). However, SQ were not correlated with reaction time on the EPT after adjusting for IQ (partial r=0.14, p=0.20, CI [-0.06, 0.32]).

**Figure 2:**
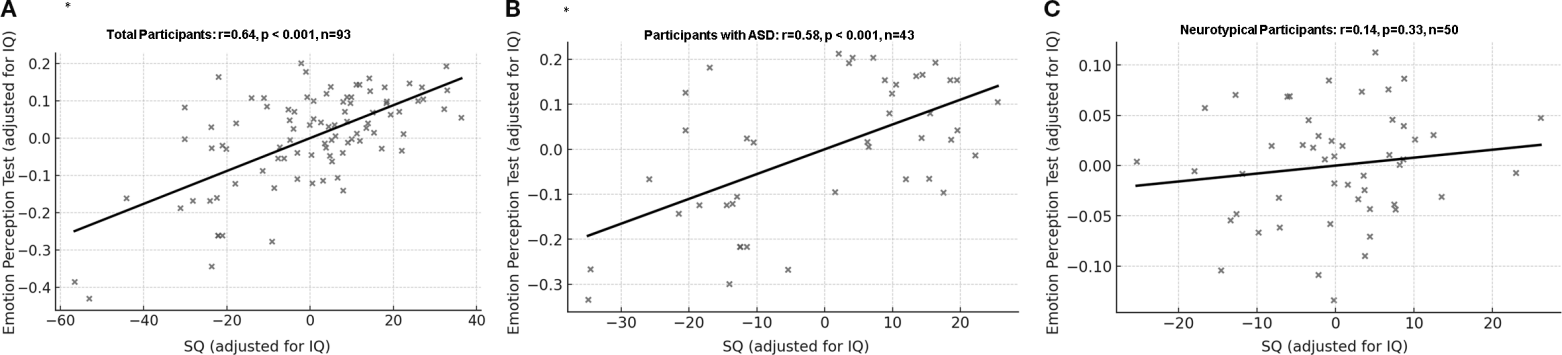
Correlation between correction rate of Emotion Perception Test and social quotient with IQ as covariate. **(A)** All participants: partial r = 0.64, df = 90, p < 0.001, 95% Confidence Interval [0.46, 0.77]. **(B)** Participants with ASD: partial r = 0.58, df = 40, p < 0.001, 95% Confidence Interval [0.33, 0.75]. **(C)** Neurotypical participants: partial r = 0.14, df = 47, p = 0.33, 95% Confidence Interval [-0.10, 0.38]. ASD, Autism spectrum disorder; SQ, Social quotient; *p=0.003 was considered significant.

For the ASD group, SQ scores were positively correlated with the EPT correction rate after adjusting for IQ (partial r=0.58, p<0.001, CI [0.33, 0.75]). However, for the control group, SQ scores were not correlated with the EPT correction rate after adjusting for IQ (r=0.14, p=0.33, CI [-0.10, 0.38]). (**Figure 2**) Correlation analysis on the whole data set also showed similar results (**Supplementary 2**).

### 3.8 Correlations between SQ and MEPT scores

For all participants, SQ scores were positively correlated with the correction rates of MEPT-1 (r=0.60, p<0.001, CI [0.39, 0.74]) and MEPT-2 (r=0.37, p<0.001, CI [0.17, 0.52]) after adjusting for IQ. However, SQ scores were not correlated with reaction times on MEPT-1 (r=0.04, p=0.71, CI [-0.23, 0.27]) and MEPT-2 (r=-0.05, p=0.62, CI [-0.24, 0.13]) after adjusting for IQ and correction for multiple comparison.

**Figure 3:**
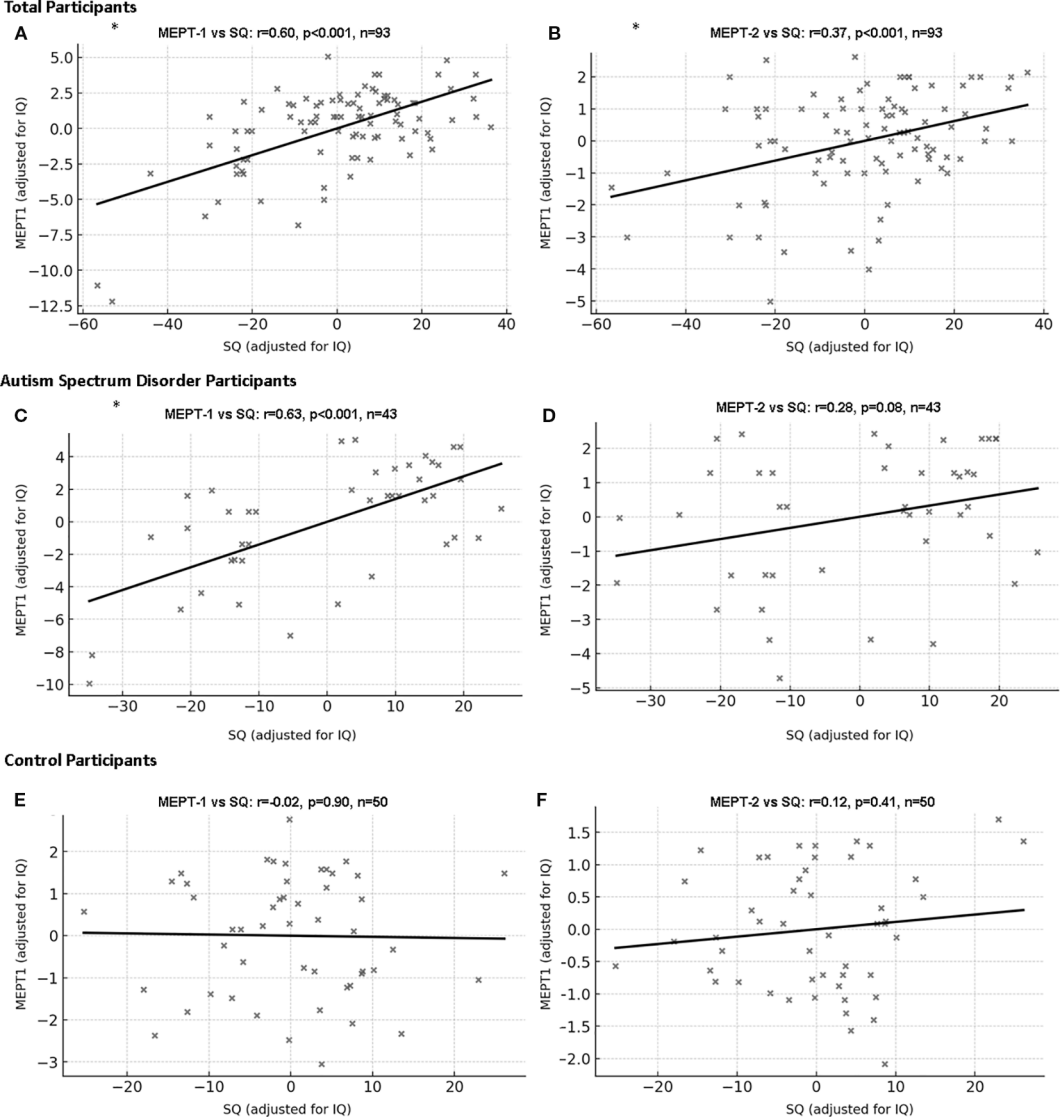
Correlation between Music Emotion Perception Test and social quotient with IQ as covariate. **(A)** MEPT-1 (all participants): partial r=0.60, df=90, p<0.001, 95% Confidence Interval [0.39, 0.74]. **(B)** MEPT-2 (all participants): partial r=0.37, df=90, p<0.001, 95% Confidence Interval [0.17, 0.52]. **(C)** MEPT-1 (participants with ASD): partial r=0.63, df=40, p<0.001, 95% Confidence Interval [0.37, 0.80]. **(D)** MEPT-2 (participants with ASD): partial r=0.28, df=40, p=0.08, 95% Confidence Interval [0.13, 0.55]. **(E)** MEPT-1 (neurotypical participants): partial r=-0.02, df=47, p=0.90, 95% Confidence Interval [-0.30, 0.26]. **(F)** MEPT-2 (neurotypical participants): partial r=0.12, df=47, p=0.41, 95% Confidence Interval [-0.20, 0.39]. ASD, Autism spectrum disorder; SQ, Social quotient; MEPT, Music emotion perception test; *p=0.003 was considered significant.

In the ASD group, SQ scores were positively correlated with MEPT-1 (r=0.63, p<0.001, CI [0.37, 0.80]) but not with MEPT-2 (r=0.28, p=0.08, CI [0.01, 0.55]) scores after adjusting for IQ and multiple comparison. In the control group, SQ scores were not correlated with MEPT-1 (r=-0.02, p=0.90, CI [-0.30, 0.26]) and MEPT-2 (r=0.12, p=0.41, CI [-0.20, 0.39]) scores (**Figure 3**). Analysis on the whole data set including intellectual disability participants also showed similar results (**Supplementary 2**).

Closer inspection found suspected ceiling effects on MEPT-3. The percentage of maximum scorers were most prominent in MEPT-3(ASD group=28.57%, control group=84.00%), when compared to MEPT-1(ASD group=1.19%, control group=32.00%) to MEPT-2(ASD group=5.95%, control group=20.00%). To account for the ceiling effect, a separate correlation analysis between MEPT-1, MEPT-2, MEPT-3 and SQ with IQ as covariate was performed excluding maximum scorers for each test. For all participants, MEPT-1, MEPT-2 and MEPT-3 was still significant. For the ASD group, MEPT-1 and MEPT-3 was significant, while MEPT-2 did not survive adjustment after multiple comparison. The control group showed non-significance in MEPT-1 and MEPT-2. Control group analysis for MEPT-3 could not be performed due to low sample size (n=8). Detailed data can be seen in the supplement (**Supplementary 4**).

## 4 Discussion

The ASD group had a lower education level and lower full-scale and subscale IQ scores (44, 45) compared to healthy controls. They also scored lower on SQ (47) and both the EPT (53) and MEPT (54). MEPT-1 and MEPT-2 demonstrated high internal consistency, while MEPT-3 was not used for inference due to high ceiling effect. We considered the minimal acceptable ordinal alpha to be 0.7 (55), and in our study, the separate ordinal alpha coefficients for each subscale of MEPT exceeded 0.85, while the total ordinal alpha coefficient of the scale was excellent and equal to 0.951. The exploratory factor analysis also revealed sound construct validity for most items on the MEPT. Item communalities below 0.4 were considered to have weak relationships with other items; thus, 6 of the 39 total items of the test will be considered for revision in future versions of the MEPT (58). The MEPT-1 and MEPT-2 also demonstrated acceptable concurrent validity, as we considered coefficient values higher than 0.5 as good and higher than 0.75 as excellent. However, MEPT-3 was deem unacceptable due to high ceiling effect (59). No reports of discomfort due to hypersensitivity towards the MEPT was found, but the possibility should be cautioned.

Low emotion perception ability is a key feature of ASD, as noted by previous studies (20, 21, 26, 27). This was shown in our research by significantly lower EPT scores in the ASD group in comparison to the control group EPT scores. Our study also found that the ASD group scored significantly worse compared to the control group on the MEPT. This was same even when removing intellectual disability participants and adjusting for IQ, although we must emphasize that even after these statistical corrections, the IQ between the groups were significantly different. Studies suggesting that people with ASD may have less deficits in recognizing emotions based on music compared to TD peers have used the shared affective motion experience model to explain this (32, 33). In the shared affective motion experience model, the auditory and motor signals stimulate the mirror neuron system and the limbic system among other areas, resulting in a shared affective motion experience in the listener (33). Molnar-Szakacs et al. (32) argue that although people with ASD have shown deficits in the mirror neuron system, the repetitive and predictable nature of music allows the mirror neuron system to be sufficiently stimulated, resulting in ASD individuals experiencing the emotions in music. However, the ASD group in this study had significantly lower IQ scores compared to the control group, which could indicate that the brain may not have had sufficient intellectual resources for such compensatory methods. We should note that 44% of the participants in the ASD group from our study had comorbid intellectual disability, which is slightly higher than recent global median values of 33~35% (60, 61).

After controlling for IQ, ASD groups still had significantly lower social quotient than the controls. Also, EPT scores and MEPT scores were significantly lower in the ASD group than the control. We believe this is in part due to the inherent difficult people with ASD have understanding emotion and social cues, while also in part due to the compensatory effect of IQ. Livingston et al. also noted that in ASD individuals, IQ is needed in compensating for difficulties in ASD (62). Since more and more ASD individuals are reported to have higher IQ than before (63), IQ should be taken into consideration when designing diagnostic or treatment methods for people with ASD.

Similar to previous research (18, 19), our study also revealed a correlation between social intelligence and emotion recognition, as correlations with EPT and SQ scores were moderate to strong for the full sample and the ASD group even after accounting for IQ (0.64 and 0.58 respectively) However, our study failed to find a significant correlation between EPT and SQ scores in the control group. We speculate that this could be due to a ceiling effect in the data. This usually happens when the test is too easy in relation to participants’ abilities (64) and has been shown in many studies, especially involving high-functioning or normally functioning groups (64–66). Researchers have used various methods to overcome this phenomenon, such as lowering the stimuli’s intensity (67) or shortening the time of exposure to the stimuli (68).

In all participants and in the ASD group, the correlations between SQ and the MEPT scores were similar to those between SQ and EPT, ranging from r = 0.37 to 0.63. In the control group, however, the correlations between MEPT and SQ were not significant. We believe the same issues noted for EPT explain this pattern, most clearly for MEPT-3. In MEPT-3, each item presents a musical excerpt and four faces; participants choose the facial expression most different from the music. During development, testers found “most different” difficult to judge, so we revised the items to include one different facial expression and three similar expressions (rather than four different expressions). This unintentionally allowed some participants to identify the odd face without listening to the music, making the task easier for the control group. Consequently, most TD participants achieved perfect scores or missed only one item on MEPT-3; 84% of the TD group scored perfectly. To address the ceiling effect, we repeated the correlations after removing perfect scorers for all tests. The results were similar: significant correlations between MEPT and SQ in the full sample and ASD participants, but non-significant correlations in TD controls. This underscores the importance of appropriate difficulty when developing tests. Although MEPT scores correlated with SQ in ASD participants, this association was not strong enough to produce a correlation in TD controls. MEPT-3 seems to be too easy for controls, and therefore, interpretation of MEPT-3 results in the current situation seems unreliable and was removed from the main analysis. Future versions should include greater difficulty variation and item recalibration.

There were no significant differences in reaction times on either the EPT or MEPT between the ASD and control groups. Additionally, there was no correlation between reaction time and SQ scores. Studies regarding reaction time in ASD groups showed mixed results, with some showing significant differences (22, 23, 28), while others showed no difference between TD groups and ASD (24, 25). We estimate that this is due to our ASD group having mostly mild to moderate autistic symptoms, with a mean cars-2 score of 29.1 (28 to 36.5 indicate mild to moderate autistic symptoms, depending on their functions). Wagener et al. (23) reported that more severe autism was correlated with longer reaction time and Fink et al. found that there were no differences in reaction time in facial emotion recognition tests between TD group and high-functioning ASD group. Since our group had less severe autistic symptoms, the difference in reaction could have been small enough to no be detected in our sample. A *post hoc* sensitivity analysis of the null results indicated that, with our total sample size (N = 134), the study would detect only between-group effects larger than approximately d = 0.45 (Cohen’s d). Accordingly, small to moderate differences between groups may have been undetectable in this study, and the null findings should be interpreted as inconclusive for effects below this threshold.

Our research is not without limitations. First, the difference of IQ between the two groups were large. Although we statistically adjusted for IQ and removed low IQ individuals for main analysis, residual IQ differences between groups may still have influenced the results, and statistical adjustments may not have been enough due to the large differences between the groups. Therefore, we caution against interpreting the results as IQ-independent “autism-specific” effects. Second, we did not assess test–retest reliability. Third, because MEPT-2 and MEPT-3 also use facial stimuli, validity comparisons with EPT may introduce method variance, and observed correlations may partly reflect the shared stimulus modality. Fourth, differences between adolescents and adults in interpreting emotions should be considered; although we tested interaction effects, future work could analyze these populations separately. Fifth, test results may vary with participant engagement; additional studies should include engagement metrics to evaluate this. Finally, because the study was conducted in the Republic of Korea with Korean participants, generalizability to other cultures may be limited given potential cultural differences in emotion.

The MEPT developed in our study demonstrated evidence of internal consistency, construct validity, and concurrent validity. MEPT scores were also significantly positively correlated with social intelligence in the full sample and the ASD group. However, the MEPT did not show a significant correlation in TD participants. Although it requires updates—particularly for MEPT-2 and MEPT-3—we believe this study shows that social intelligence is associated with music emotion recognition, as evidenced by moderate correlations. We would like to again caution against generalizing these results as IQ differences were significant, and should be reanalyzed in a separate study design to truly see the pure correlations between social intelligence and music emotion recognition. We also note that, because this is a cross-sectional study, we cannot infer causality or establish predictive validity. Future versions that incorporate non-music cues and a wider range of difficulty may improve the program.

## Data Availability

The datasets presented in this article are not readily available because data will be provided after approval from the institutional review board. Requests to access the datasets should be directed to hduk70@gmail.com.
