# Supplementary material for: Recognizing emotions in music through a computerized method: a novel way of evaluating social maturity
Source: Front Psychiatry. 2025 Oct 17;16:1674615. doi: 10.3389/fpsyt.2025.1674615 (PMC12576337; doi:10.3389/fpsyt.2025.1674615)
Supplement: Supplementary file 1 [file Table1.docx]

| MEPT1 | | MEPT2 | | MEPT3 | |
| --- | --- | --- | --- | --- | --- |
| Variables | Loading | Variables | Loading | Variables | Loading |
| MEPT1-1 | 0.405 | MEPT2-1 | 0.902 | MEPT3-1 | 0.859 |
| MEPT1-2 | 0.631 | MEPT2-2 | 0.639 | MEPT3-2 | 0.692 |
| MEPT1-3 | 0.745 | MEPT2-3 | 0.487 | MEPT3-3 | 0.968 |
| MEPT1-4 | 0.511 | MEPT2-4 | 0.764 | MEPT3-4 | 0.957 |
| MEPT1-5 | 0.542 | MEPT2-5 | 0.633 | MEPT3-5 | 0.847 |
| MEPT1-6 | 0.614 | MEPT2-7 | 0.792 | MEPT3-6 | 0.756 |
| MEPT1-7 | 0.587 | MEPT2-8 | 0.786 | MEPT3-7 | 0.944 |
| MEPT1-9 | 0.788 |  |  | MEPT3-8 | 0.885 |
| MEPT1-10 | 0.671 |  |  | MEPT3-9 | 0.888 |
| MEPT1-11 | 0.763 |  |  |  |  |
| MEPT1-13 | 0.727 |  |  |  |  |
| MEPT1-14 | 0.519 |  |  |  |  |
| MEPT1-16 | 0.637 |  |  |  |  |
| MEPT1-17 | 0.822 |  |  |  |  |
| MEPT1-18 | 0.527 |  |  |  |  |
| MEPT1-19 | 0.537 |  |  |  |  |
| MEPT1-20 | 0.561 |  |  |  |  |
| Eigen value | 6.799 |  | 3.689 |  | 6.821 |
| Variance Explained | 42.50% |  | 52.69% |  | 75.79% |

**Supplement 1. Factor loading of Music emotional perception test**
