# Supplementary material for: Recognizing emotions in music through a computerized method: a novel way of evaluating social maturity
Source: Front Psychiatry. 2025 Oct 17;16:1674615. doi: 10.3389/fpsyt.2025.1674615 (PMC12576337; doi:10.3389/fpsyt.2025.1674615)

**Supplement 2. Analysis of ASD participants without removing intellectual disability participants.**

**Supplement Table 2-1. Differences between social maturity, EPT and MEPT**

| Variables | ASD  (n=84) | Control  (n=50) | Statistical value | 95% Confidence interval |
| --- | --- | --- | --- | --- |
| Social quotient, raw score (Mean ± SD) | 62.81±15.52 | 105.77±10.30 | t=19.23 p<0.001 d=3.44 | [38.54, 47.38] |
| Emotion Perception Test |  |  |  |  |
| Correction rate, score/total question (Mean ± SD) ^*^ | 0.65±0.14 | 0.84±0.06 | t=11.10 p<0.001 d=1.98 | [0.15, 0.22] |
| Reaction time, millisecond (Mean ± SD) | 3885.75±1369.42 | 4108.39±1024.02 | t=1.00 p=0.32 d=0.18 | [-219.85, 665.14] |
| Music Emotion Perception Test |  |  |  |  |
| MEPT-1 correction rate, raw score (Mean ± SD) ^*^ | 13.80±3.57 | 18.30±1.57 | t=10.05 p<0.001 d=1.80 | [3.62, 5.39] |
| MEPT-1 reaction time, millisecond (Mean ± SD) | 2834.18±1840.36 | 2425.48±1609.40 | t=-1.30 p=0.20 d=0.23 | [-1029.91, 212.50] |
| MEPT-2 correction rate, raw score (Mean ± SD) ^*^ | 5.51±2.23 | 7.48±1.05 | t=6.90 p<0.001 d=1.23 | [1.40, 2.53] |
| MEPT-2 reaction time, millisecond (Mean ± SD) | 3845.50±2154.84 | 3343.13±1645.78 | t=-1.42 p=0.16 d=0.25 | [-1202.38, 197.64] |
| MEPT-3 correction rate, raw score (Mean ± SD) ^*^ | 6.05±2.97 | 8.84±0.37 | t=8.51 p<0.001 d=1.57 | [2.14, 3.45] |
| MEPT-3 reaction time, millisecond (Mean ± SD) | 2505.66±1300.91 | 2298.42±1181.77 | t=-0.92 p=0.36 d=0.16 | [-651.72, 237.25] |

*Note*. ASD: Autism spectrum disorder; SD: Standard deviation; EPT: Emotion perception test; MEPT: Music emotion perception test; d: cohen’s d; *: p=0.003 was considered significant; Independent t-test was done.

**Supplement Table 2-2. Differences between social maturity, EPT and MEPT with IQ as covariate**

| Variables | Adjusted ASD  (n=84) | Adjusted Control  (n=50) | Statistical value | 95% Confidence interval |
| --- | --- | --- | --- | --- |
| Social quotient |  |  | F=173.09 p<0.001 partial η²=0.73 |  |
| IQ = 85.00 (50^th^)  (Mean ± Standard Error) ^*^ | 67.17±1.70 | 101.16±3.49 | Δ=33.99 SE=3.88 p<0.001 | [26.31, 41.68] |
| IQ = 105.00 (75^th^)  (Mean ± Standard Error) ^*^ | 73.72±2.79 | 105.29±1.84 | Δ=31.57 SE=3.34 p<0.001 | [24.96, 38.17] |
| Emotion Perception Test |  |  | F=44.68 p<0.001 partial η² =0.41 |  |
| IQ = 85.00 (50^th^)  (Mean ± Standard Error) ^*^ | 0.66±0.02 | 0.81±0.03 | Δ=0.15 SE=0.03 p<0.001 | [0.08, 0.22] |
| IQ = 105.00 (75^th^)  (Mean ± Standard Error) ^*^ | 0.69±0.02 | 0.83±0.02 | Δ=0.15 SE=0.03 p<0.001 | [0.09, 0.21] |
| Music Emotion Perception Test-1 |  |  | F=45.33 p<0.001 partial η² =0.41 |  |
| IQ = 85.00 (50^th^)  (Mean ± Standard Error) ^*^ | 14.52±0.38 | 17.23±0.78 | Δ=2.72 SE=0.87 p=0.002 | [1.00, 4.43] |
| IQ = 105.00 (75^th^)  (Mean ± Standard Error) ^*^ | 15.60±0.62 | 18.19±0.41 | Δ=2.59 SE=0.74 p=0.001 | [1.12, 4.06] |
| Music Emotion Perception Test-2 |  |  | F=22.46 p<0.001 partial η² =0.26 |  |
| IQ = 85.00 (50^th^)  (Mean ± Standard Error) | 5.83±0.24 | 6.71±0.50 | Δ=0.88 SE=0.56 p=0.12 | [-0.22, 1.98] |
| IQ = 105.00 (75^th^)  (Mean ± Standard Error) | 6.31±0.40 | 7.40±0.26 | Δ=1.09 SE=0.48 p=0.02 | [0.15, 2.04] |
| Music Emotion Perception Test-3 |  |  | F=24.60 p<0.001 partial η² =0.27 |  |
| IQ = 85.00 (50^th^)  (Mean ± Standard Error) ^*^ | 6.54±0.31 | 8.79±0.63 | Δ=2.25 SE=0.70 p=0.002 | [0.87, 3.64] |
| IQ = 105.00 (75^th^)  (Mean ± Standard Error) | 7.27±0.50 | 8.84±0.33 | Δ=1.56 SE=0.60 p=0.01 | [0.37, 2.75] |

*Note*. ASD: Autism spectrum disorder; EPT: Emotion perception test; MEPT: Music emotion perception test; Δ : Mean difference between group; SE: Standard error; *: p=0.003 was considered significant; Univariate ANCOVA with IQ as the covariate was also performed. In the case the group x IQ interaction was significant, conditional group differences with linear regression models including Group, IQ, and Group × IQ, re-centering IQ at prespecified values within the observed overlap of the two groups’ IQ distributions (50th percentile = 85.00 and 75th percentile = 105) was noted

**Supplement Figure 2-3. IQ distribution between group**

Distribution graph of the IQ density between ASD and control group. IQ distributions 50^th^ percentile (85.00) and 75^th^ percentile (105.00) were chosen for points of analysis since they were in the overlapping zone.

*Note*. ASD: Autism spectrum disorder


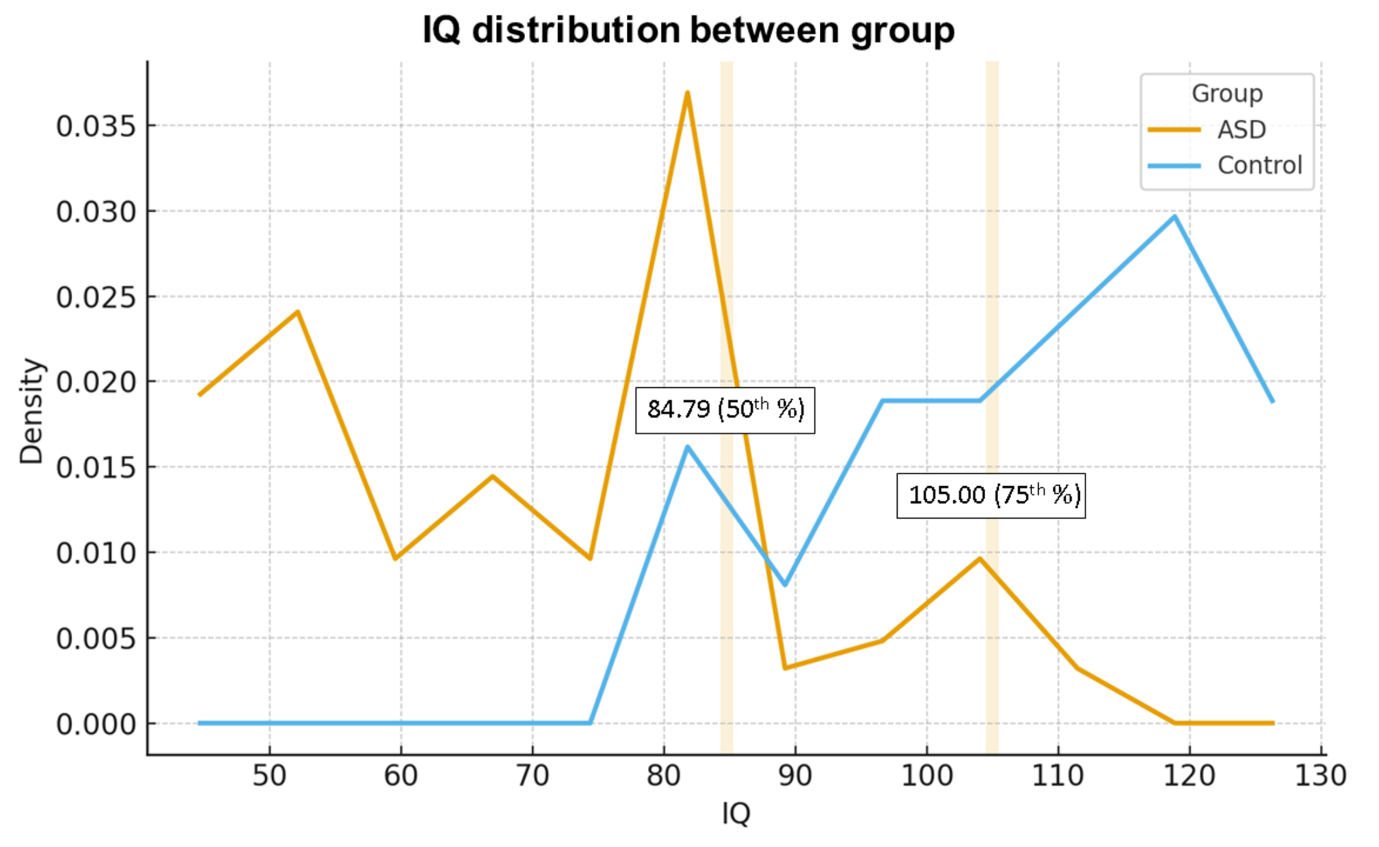


**Supplement Figure 2-4. Comparison of Adjusted SQ, EPT and MEPT between ASD group and control group with IQ as covariate**

A: Adjusted social quotient for ASD and control group at IQ=85.00 and IQ=105.00: F=173.09, p<0.001, partial η²=0.73, Mean ± Standard Error for ASD at IQ 84.79=67.17±1.70, Mean ± Standard Error for Control at IQ 84.79=101.16±3.49, Mean ± Standard Error for ASD at IQ 105.00=73.72±2.79, Mean ± Standard Error for Control at IQ 105.00=105.29±1.84

B: Adjusted Emotion Perception Test correction rate for ASD and control group at IQ=85.00 and IQ=105.00: F=44.68, p<0.001, partial η² =0.41, Mean ± Standard Error for ASD at IQ 84.79=0.66±0.02, Mean ± Standard Error for Control at IQ 84.79=0.81±0.03, Mean ± Standard Error for ASD at IQ 105.00=0.69±0.02, Mean ± Standard Error for Control at IQ 105.00=0.83±0.02

C: Adjusted Music Emotion Perception Test-1 score for ASD and control group at IQ=85.00 and IQ=105.00: F=45.33, p<0.001, partial η² =0.41, Mean ± Standard Error for ASD at IQ 84.79=14.52±0.38, Mean ± Standard Error for Control at IQ 84.79=17.23±0.78, Mean ± Standard Error for ASD at IQ 105.00=15.60±0.62, Mean ± Standard Error for Control at IQ 105.00=18.19±0.41

D: Adjusted Music Emotion Perception Test-2 score for ASD and control group at IQ=85.00 and IQ=105.00: F=22.46, p<0.001, partial η² =0.26, Mean ± Standard Error for ASD at IQ 84.79=5.83±0.24, Mean ± Standard Error for Control at IQ 84.79=6.71±0.50, Mean ± Standard Error for ASD at IQ 105.00=6.31±0.40, Mean ± Standard Error for Control at IQ 105.00=7.40±0.26

E: Adjusted Music Emotion Perception Test-3 score for ASD and control group at IQ=85.00 and IQ=105.00: F=24.60, p<0.001, partial η² =0.27, Mean ± Standard Error for ASD at IQ 84.79=6.54±0.31, Mean ± Standard Error for Control at IQ 84.79=8.79±0.63, Mean ± Standard Error for ASD at IQ 105.00=7.27±0.50, Mean ± Standard Error for Control at IQ 105.00=8.84±0.33

*Note*. ASD: Autism spectrum disorder; SQ: Social quotient; EPT: Emotion perception test; MEPT: Music emotion perception test; *: p=0.003 was considered significant


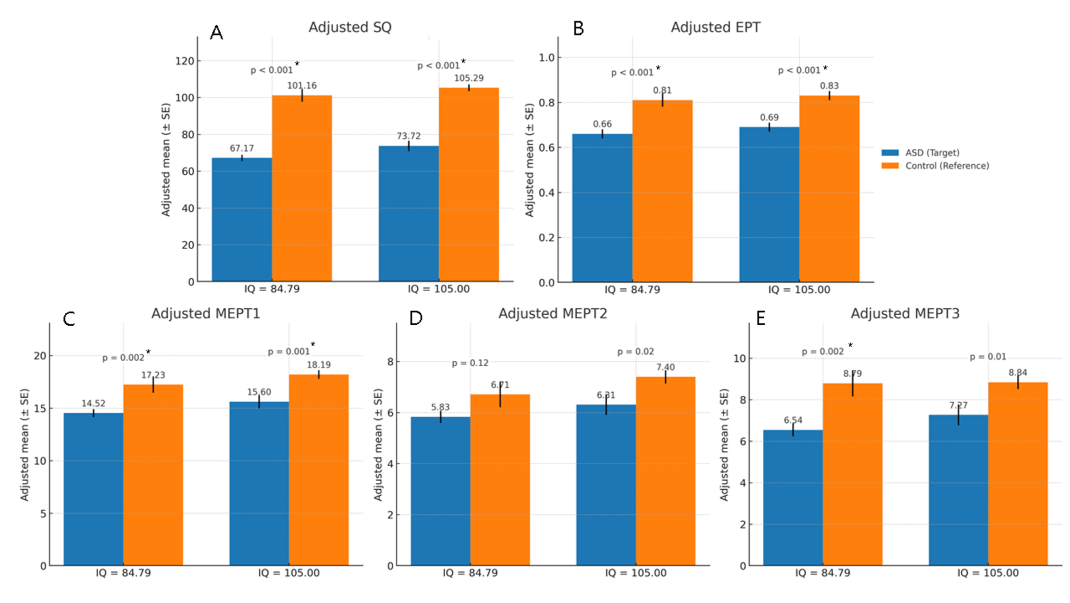


**Supplement Figure 2-5. Correlation between correction rate of Emotional Perception Test and social quotient with IQ as covariate**

A: All participants: r = 0.60, df = 131, p < 0.001, 95% Confidence Interval [0.44, 0.71]

B: Participants with ASD: r = 0.54, df = 81, p < 0.001, 95% Confidence Interval [0.35, 0.68]

C: Neurotypical participants: r = 0.14, df = 47, p = 0.33, 95% Confidence Interval [-0.10, 0.38]

*Note*. ASD: Autism spectrum disorder; SQ: Social quotient; *: p=0.003 was considered significant


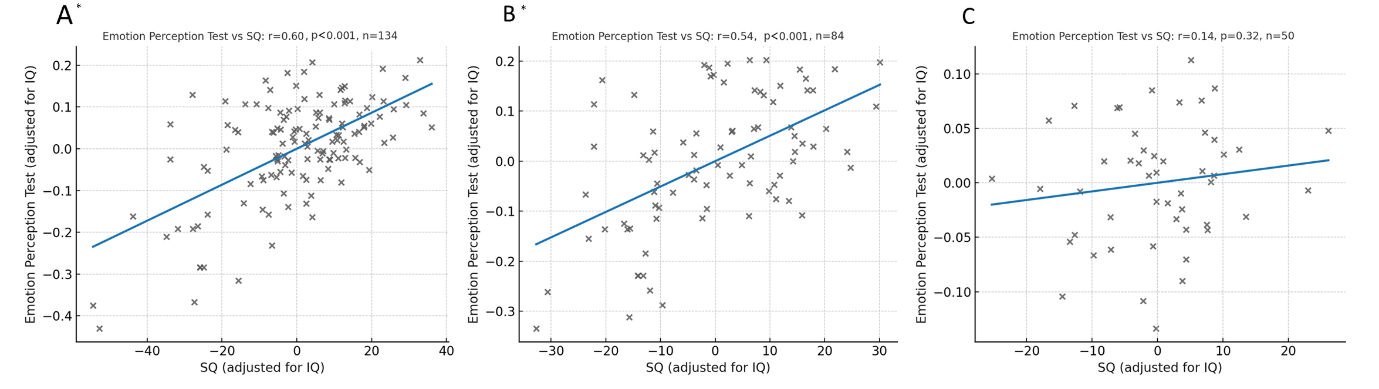


**Supplement Figure 2-6. Correlation between Music Emotion Perception Test and social quotient with IQ as covariate**

A: MEPT-1 (all participants): r=0.52, df=131, p<0.001, 95% Confidence Interval [0.32, 0.66]

B: MEPT-2 (all participants): r=0.33, df=131, p<0.001, 95% Confidence Interval [0.17, 0.46]

C: MEPT-3 (all participants): r=0.41, df=131, p<0.001, 95% Confidence Interval [0.25, 0.57]

D: MEPT-1 (participants with ASD): r=0.54, df=81, p<0.001, 95% Confidence Interval [0.56, 0.81]

E: MEPT-2 (participants with ASD): r=0.30, df=81, p=0.005, 95% Confidence Interval [0.28, 0.61]

F: MEPT-3 (participants with ASD): r=0.40, df=81, p<0.001, 95% Confidence Interval [0.30, 0.66]

G: MEPT-1 (neurotypical participants): r=-0.02, df=47, p=0.90, 95% Confidence Interval [-0.30, 0.26]

H: MEPT-2 (neurotypical participants): r=0.12, df=47, p=0.41, 95% Confidence Interval [-0.20, 0.39]

I: MEPT-3 (neurotypical participants): r = -0.32, df = 47, p = 0.02, 95% Confidence Interval [-0.53, -0.09]

*Note*. ASD: Autism spectrum disorder; SQ: Social quotient; MEPT: Music emotion perception test; *: p=0.003 was considered significant


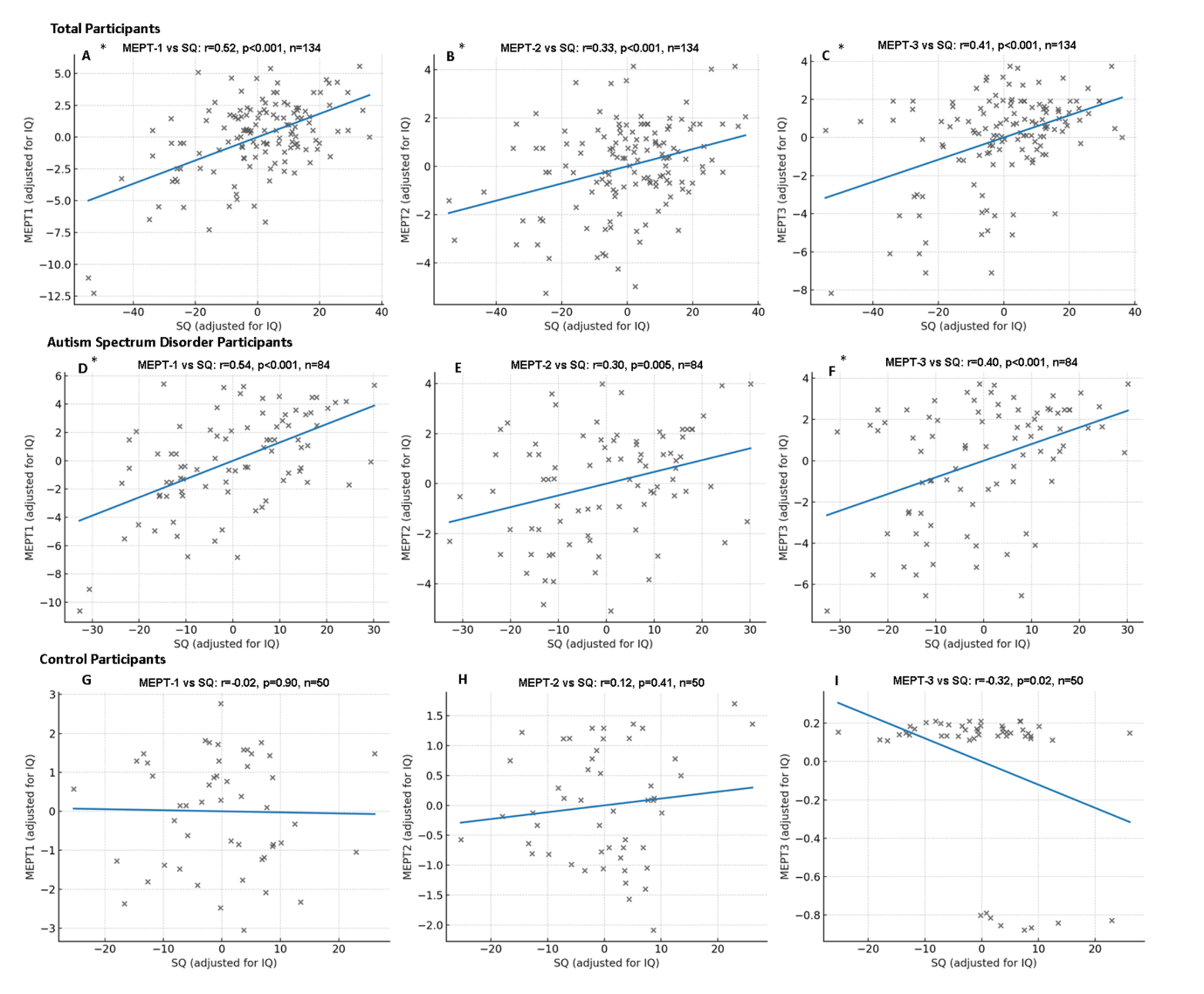

Supplement: Supplementary file 2 [file Table2.docx]
