# Supplementary material for: Recognizing emotions in music through a computerized method: a novel way of evaluating social maturity
Source: Front Psychiatry. 2025 Oct 17;16:1674615. doi: 10.3389/fpsyt.2025.1674615 (PMC12576337; doi:10.3389/fpsyt.2025.1674615)
Supplement: Supplementary file 3 [file Table3.docx]

**Supplement 3. Age group interactions between ASD group and control group for SQ, EPT and MEPT scores**

**Supplement Table 3-1. Number of adolescents and adults per group**

| Age group | ASD  (n=84) | Control  (n=50) | Statistical value |
| --- | --- | --- | --- |
| Adolescent (13 ~ 18 years old) | 29 (34.5%) | 10 (20.0%) | χ^2^=3.20 p=0.07 |
| Adult (19 ~ 33 years old) | 55 (65.5%) | 40 (80.0%) |  |

*Note*. ASD: Autism spectrum disorder; Chi-square was used for analysis.

**Supplement 3-2. Differences between SQ, EPT and MEPT controlling for IQ and Age group.**

| Variables | Adjusted ASD  (n=84) | Adjusted Control  (n=50) | Statistical value | 95% Confidence interval |
| --- | --- | --- | --- | --- |
| Social quotient |  |  | F=97.61 p<0.001 partial η²=0.43 |  |
| Interaction between Group x Age group |  |  | F=0.97 p=0.33 |  |
| IQ = 85.00 (50^th^)  (Mean ± Standard Error) | 66.69±1.69 | 99.51±2.53 | Δ=32.82 SE=3.32 p<0.001 | [26.25, 39.40] |
| Emotion Perception Test |  |  | F=28.07 p<0.001 partial η² =0.18 |  |
| Interaction between Group x Age group |  |  | F=1.41 p=0.24 |  |
| IQ = 85.00 (50^th^)  (Mean ± Standard Error) | 0.66±0.02 | 0.81±0.02 | Δ=0.15 SE=0.03 p<0.001 | [0.10, 0.21] |
| Music Emotion Perception Test-1 |  |  | F=13.89 p<0.001 partial η² =0.10 |  |
| Interaction between Group x Age group |  |  | F=0.21 p=0.65 |  |
| IQ = 85.00 (50^th^)  (Mean ± Standard Error) | 14.57±0.38 | 17.32±0.56 | Δ=2.76 SE=0.74 p<0.001 | [1.29, 4.22] |
| Music Emotion Perception Test-2 |  |  | F=4.76 p=0.03 partial η² =0.04 |  |
| Interaction between Group x Age group |  |  | F=0.01 p=0.91 |  |
| IQ = 85.00 (50^th^)  (Mean ± Standard Error) | 5.86±0.24 | 6.90±0.36 | Δ=1.04 SE=0.48 p=0.03 | [0.10, 1.98] |
| Music Emotion Perception Test-3 |  |  | F=9.55 p=0.002 partial η² =0.07 |  |
| Interaction between Group x Age group |  |  | F=0.05 p=0.82 |  |
| IQ = 85.00 (50^th^)  (Mean ± Standard Error) | 6.51±0.31 | 8.37±0.46 | Δ=1.86 SE=0.60 p=0.002 | [0.67, 3.05] |

*Note*. ASD: Autism spectrum disorder; EPT: Emotion perception test; MEPT: Music emotion perception test; Δ : Mean difference between group; SE: Standard error; The homogeneity of regression slopes was non-significant for all interactions with IQ as covariate. Controlling for IQ, a two-way ANCOVA was performed between Age group (Adolescent vs Adult) and Group (ASD vs Control)
