# Supplementary material for: Recognizing emotions in music through a computerized method: a novel way of evaluating social maturity
Source: Front Psychiatry. 2025 Oct 17;16:1674615. doi: 10.3389/fpsyt.2025.1674615 (PMC12576337; doi:10.3389/fpsyt.2025.1674615)

**Supplement 4. Analysis of correlation between SQ and MEPT after removing ceiling effects**

**Supplement Table 4-1. Number of maximum scorers per group**

| Test name | ASD  (n=84) | Control  (n=50) | Total  (n=134) |
| --- | --- | --- | --- |
| Music Emotion Perception Test -1 | 1 (1.19%) | 16 (32.0%) | 17 (12.69%) |
| Music Emotion Perception Test -2 | 5 (5.95%) | 10 (20.0%) | 15 (11.19%) |
| Music Emotion Perception Test -3 | 24 (28.57%) | 42 (84.0%) | 66 (49.25%) |

*Note*. ASD: Autism spectrum disorder

**Supplement Figure 4-2. Correlation between Music Emotion Perception Test and social quotient with IQ as covariate and after removing maximum scorers**

A: MEPT-1 (all participants): n=117, partial r=0.51, df=114, p<0.001, 95% Confidence interval [0.32, 0.66]

B: MEPT-2 (all participants): n=119, partial r=0.29, df=116, p<0.001, 95% Confidence interval [0.13, 0.45]

C: MEPT-3 (all participants): n=68, partial r=0.45, df=65, p<0.001, 95% Confidence interval [0.22, 0.63]

D: MEPT-1 (participants with ASD): n=83, partial r=0.54, df=80, p<0.001, 95% Confidence interval [0.33, 0.69]

E: MEPT-2 (participants with ASD): n=79, partial r=0.27, df=76, p=0.02, 95% Confidence interval [0.06, 0.47]

F: MEPT-3 (participants with ASD): n=60, partial r=0.38, df=57, p=0.003, 95% Confidence interval [0.11, 0.59]

G: MEPT-1 (neurotypical participants): n=34, partial r=-0.10, df=31, p=0.57, 95% Confidence interval [-0.40, 0.22]

H: MEPT-2 (neurotypical participants): n=40, partial r=-0.02, df=37, p=0.91, 95% Confidence interval [-0.28, 0.24]

I: MEPT-3 (neurotypical participants): n=8, correlation was not performed due to low sample size


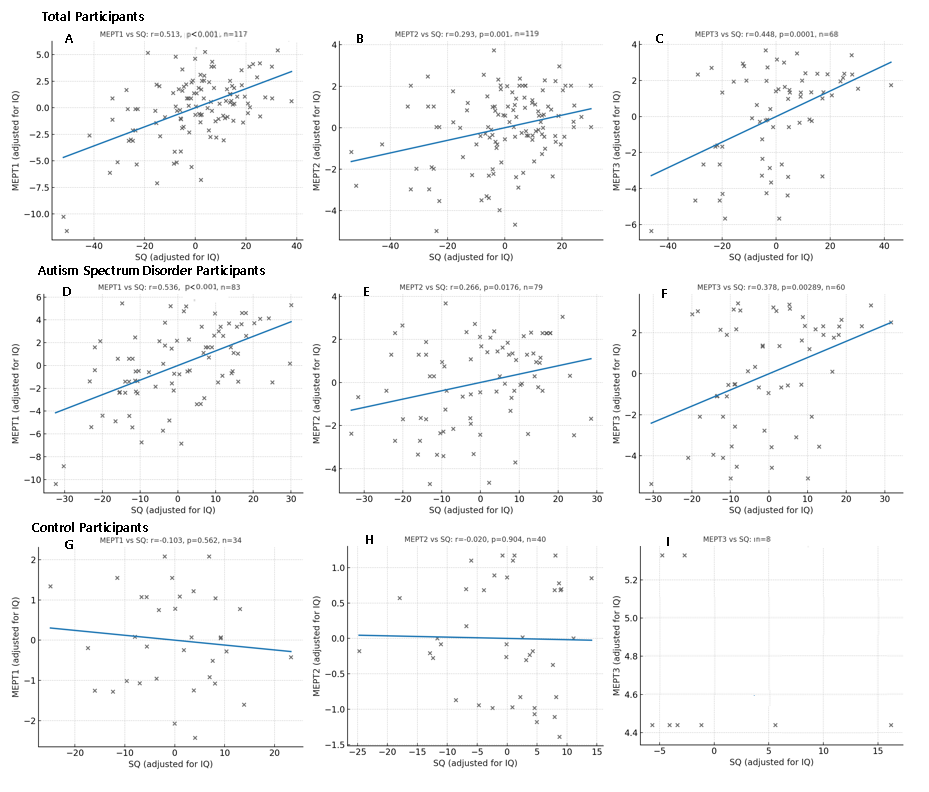

Supplement: Supplementary file 4 [file Table4.docx]
